# Physical Function, Self-Perceived Physical Fitness, Falls, Quality of Life and Degree of Disability According to Fear and Risk of Falling in Women with Fibromyalgia

**DOI:** 10.3390/jfmk9030174

**Published:** 2024-09-23

**Authors:** Ángel Denche-Zamorano, Damián Pereira-Payo, Daniel Collado-Mateo, José Carmelo Adsuar-Sala, Pablo Tomas-Carus, Jose Alberto Parraca

**Affiliations:** 1 Promoting a Healthy Society Research Group (PHeSO), Faculty of Sport Sciences, University of Extremadura, 10003 Caceres, Spain; 2 Departamento de Desporto e Saúde, Escola de Saúde e Desenvolvimento Humano, Universidade de Évora, 7004-516 Evora, Portugaljparraca@uevora.pt (J.A.P.); 3 Health Economy Motricity and Education (HEME), Faculty of Sport Sciences, University of Extremadura, 10003 Caceres, Spain; 4 Centre for Sport Studies, Rey Juan Carlos University, 28943 Madrid, Spain; 5 CIPER, Faculty of Human Kinetics, University of Lisbon, 1649-004 Lisbon, Portugal; 6 Comprehensive Health Research Centre (CHRC), University of Evora, 7004-516 Evora, Portugal

**Keywords:** exercise, balance, HQRoL, physical activity, FMS

## Abstract

**Background:** People with fibromyalgia (FM) experience a range of symptoms (chronic widespread pain, fatigue, mood disorder, sleep problems, muscle stiffness) that promote deterioration of physical condition and function. With impaired physical function, fear of falling and risk of falling increases. This study evaluated physical function, self-perceived physical fitness, falls, quality of life, and the degree of disability caused by FM according to fear and risk of falling in Spanish adult women with FM. **Methods:** Cross-sectional study involving 84 Spanish adult women with FM. Participants completed tests to assess their physical function and completed questionnaires to evaluate self-perceived physical fitness, falls, the disabling effect of FM, quality of life, fear of falling, and risk of falling. Nonparametric statistical tests were used to analyze possible intergroup differences (Mann–Whitney U test) and correlations between variables (Spearman’s Rho). **Results:** Women with a fear of falling and at risk of falling presented a worse performance in physical tests, worse self-perceived physical fitness, greater number of falls, lower quality of life, and greater degree of disability due to FM. Weak and moderate correlations were found for fear of falling and fall risk and the variables of interest. However, no intergroup differences were found, nor significant correlations in all variables. **Conclusions:** Women with FM who present fear of falling and risk of falling tend to have worse performance in physical function tests, in addition to worse self-perceived physical fitness, higher number of falls, poorer quality of life, and greater disabling effect of FM.

## 1. Introduction

Fibromyalgia (FM) is a rheumatic disease whose main symptomatology is widespread chronic pain, fatigue, sleep disturbances, and functional symptoms [1,2]. Besides being considered a musculoskeletal condition [2], FM can have some significant implications for mental health [3,4]. In fact, the pathogenesis of this disease is conditioned by physical and physiological factors, such as the body’s ability to cope with physical stress, alterations in muscle physiology, hormonal factors, and inflammatory markers, but also by emotional-cognitive factors, personal experiences, and genetic predisposition [2,5]. Although this pathology affects both men and women, the prevalence is much higher in women [6]. Age is also a risk factor, with a higher prevalence of this pathology in older people [2].

The incidence of widespread pain, with some episodes of increased somatosensory sensitivity [7], can make it difficult for FM sufferers to lead a regular life and can even be disabling for some professional activities [8]. These difficulties may end up limiting the life of the FM patient with marked losses of autonomy [9], and can even affect the patient’s social relationships. Thus, it is common for FM patients to suffer a decrease in their quality of life [10], and to have a negative perception of their own health due to the disease [11].

FM is associated with increased fall incidence and with greater fall risk and fear of falling [12,13]. Due to its marked physical symptomatology, with generalized pain and pain in specific areas being common, FM can have a major impact on the patient’s life, limiting their mobility and their ability to perform activities of daily living [7,14]. These physical limitations, such as gait difficulties or even gait impairment, are associated with an increased fall risk [15]. In addition, patients with FM, due to the affectation of the pathology and the negative impact that this disease has on mental health, may have a worse perception of their own mobility and walking capabilities, which can be reflected in an increased fear of falling, that has been shown to be more prevalent in FM patients than in non-sufferers [16].

Both fear of falling and fall risk are relevant factors in people with FM. Firstly, because the number of falls in patients with FM is associated with the severity of the disease [17]. Secondly, both fear of falling and fall risk have been shown to be associated with a variety of variables that go from the patient’s quality of life to their physical condition and also the disabling effect of the disease [18]. Thus, in sufferers of this disease, evidence supports the existence of associations of fear of falling with health-related quality of life, perceived balance difficulties, disability due to the disease, reduced performance in mobility assessment tests, and number of falls [15,16]. For its part, increased fall risk in FM patients has been shown to be associated with the severity of the disease, poor performance in physical fitness tests, and even social isolation [19,20].

Thus, the existing evidence suggests that FM patients who also have increased fall risk or fear of falling may be more vulnerable in terms of loss of autonomy, ability to perform activities of daily living, well-being, and overall health. Therefore, the objectives of this study were to analyze physical function, perceived physical fitness, falls, health-related quality of life, perceived health status, and the degree of disability due to the disease in women with FM according to fear of falling and fall risk. The hypotheses of this study were:

(1) Women with FM with fear of falling have worse physical test performance, a higher number of previous falls, worse perceived physical condition, worse health-related quality of life, worse self-perceived health, and a greater degree of disability due to the disease than women without fear of falling.
(2) Women with FM at risk of falls have worse performance on physical tests, a higher number of previous falls, worse perceived physical condition, worse health-related quality of life, worse self-perceived health, and a greater degree of disability due to the disease than women without risk of falls.
(3) There are correlations between physical function, perceived physical condition, previous falls, health-related quality of life, self-perceived health, and degree of disability due to the disease with fear of falling (assessed with the FES-I) and risk of falling (assessed with the ABC Scale).

## 2. Materials and Methods

### 2.1. Design and Participants

This was a descriptive cross-sectional study of Spanish adult women with FM. Participants were recruited through local and national FM associations. The recommendations of the STROBE guidelines (Supplementary Table S1) for cross-sectional studies were followed [21]. The inclusion criteria were: being a woman over 18 years of age, having been diagnosed with FM by a rheumatologist and meeting the diagnostic criteria of the American College of Rheumatology [22], having the capacity to perform the physical tests of the study, and reading, understanding and accepting the informed consent of the study. Exclusion criteria were: (1) suffering from a pathology that contraindicates the practice of exercise (the physical activity readiness questionnaire (PAR-Q) was administered [23]); (2) suffering from a pathology requiring the use of psychotropic drugs; (3) suffering from any neurodegenerative pathology such as Parkinson’s, Multiple Sclerosis or similar; (4) not being able to complete the physical fitness tests and/or not completing the study questionnaires.

The initial sample consisted of 96 women, although 12 of them did not meet the inclusion criteria as they did not complete all the tests and questionnaires. The final sample consisted of 84 adult women with FM from four Spanish locations: Cáceres, Elche, Madrid, and Palencia. This study was approved by the Bioethics and Biosafety Committee of the University of Extremadura (approval number: 79/2018) following the ethical guidelines of the Declaration of Helsinki.

### 2.2. Procedures

All the procedures described below were carried out by the participants between January and April 2022, both the completion of the questionnaires and the physical tests.

Participants were required to complete a questionnaire with socio-demographic questions including variables such as age, civil status, employment situation, smoking status, drinking status, years since FM diagnosis, years with FM symptoms, and number of falls.

*Physical test*

Time Up and Go (TUG): This test was performed in its 3-meter format, which has been shown to be valid and reliable (ICC = 0.93) [24]. This test has been proven to be related to the risk of falling in older adults [25]. Participants began sitting in a chair and, at the ready signal, had to get up from the chair, walk a distance of 3 m, make a 180° turn, return to the chair, and sit down. The time to complete the whole process was timed. This test was also performed in its imagined version (i-TUG), at the voice of ready and go, the participant had to visualize that she was performing the test in an imagined way and say “Go” at the moment she mentally completed it. In addition, the test was performed in a dual task version (Dual TUG), the participants had to perform the test while counting down two by two from the number 50. With the results in TUG and i-TUG, the Delta TUG ratio, a measure related to the risk of falling, was calculated with the formula: [(Time in TUG realized − Time in i-TUG/(Time in TUG realized − Time in i-TUG)/2)] ∗ 100 [25].

Four Step Square Test (FSST): This test is used to assess dynamic stability and coordination. Four squares numbered 1 to 4 were made with adhesive tape on the floor. The participants started at square number 1, faced square number 2, and as fast as possible, when they were given the go signal, they had to move from square number 1 to square numbers 2, 3, and 4 and back to square 3, 2 and 1 without touching the adhesive tapes and with both feet touching the floor before changing squares. This test was found to be valid and reliable (ICC = 0.98) [26,27]. Two measurements were made, and the best result was taken as valid.

Walking speed. Two walking speed tests were performed: 4 m Walking Test (4 m Walking Test, 4mWT) [28] and 30 m Walking Test (30 m Walking Test, 30mWT) [29], both have been shown to be valid and reliable tests (ICC = 0.97 and ICC = 0.93, respectively). In a flat, unobstructed corridor, participants had to walk the indicated distances at maximum walking speed. Two measurements were performed, and the best result was taken as the valid one.

Six min Walking Test (6mWT): This test measures the maximum distance participants could walk in 6 min. A rectangular circuit of 45.7 m was performed [30]. This test was found to be valid and reliable (ICC = 0.92, SEM = 23.5, MDC = 65.2) [24].

The 30” Sit to Stand: This test assesses lower body strength. The test consisted of counting the number of times the participants managed to sit down and stand up from a chair within 30 s. Participants began sitting in the chair with their backs against the backrest, arms crossed at the chest, and feet flat on the floor. This test was shown to be valid and reliable (ICC = 0.91, SEM = 0.91, MDC = 2.52) [31]. The test was also performed in a dual task version (Dual Sit to Stand), participants had to perform the test while counting down 2 by 2 from the number 100. The test was also performed in its 5 repetitions version (5 reps Sit to Stand), timing the time it took participants to perform 5 repetitions to the go signal.

Maximum Handgrip Strength and Strength/Weight Ratio: Handgrip strength was assessed (ICC = 0.95, SEM = 1.46, MDC = 4.04) using a TKK 5101 Grip-D digital dynamometer; Takey, Tokyo, Japan [32]. Two measurements were taken per hand. The best performance was taken to determine the Strength/Weight Ratio, the maximum force of each participant was divided by her weight.

The 30” Biceps Curl Test: Test evaluates the strength of the upper limbs. It consisted of counting the number of repetitions that the participants could perform, lifting a 2 kg dumbbell in flexion extension of the arm in a 30 s frame. This test was found to be valid and reliable (ICC= 0.92, SEM = 1.1, MDC = 3.2) [31].

Sit and Reach: Test to assess lower limb flexibility. The chair sit and reach test is a valid and reliable test in women with FM (ICC = 0.94, SEM = 3.1, MDC = 8.7) [24]. Seated in a chair with one leg extended, the participant had to lean forward and slide her hands down the extended leg in an attempt to reach or exceed the tip of her foot. Missing or excess centimeters to the toe were recorded. Negative values indicated that the participant did not reach the toe. Two measurements were taken per limb, taking the best value of the four measurements.

Back Scratch: A test to assess the flexibility of the upper limbs, measuring the total range of motion of the shoulder. It is a valid and reliable test in women with FM (ICC = 0.96, SEM = 2.8, MDC = 7.7) [24]. The distance between the fingertips was measured when trying to bring them together behind the back. Missing centimeters (negative score) or excess centimeters (positive score) to bring the two fingertips together were recorded. Two measurements were made per side, and the best value was taken.

*Questionnaires*

Self-Reported Fitness: This was assessed with the International Fitness Scale (IFIS). An instrument composed of five Likert-scale items on the participants’ perceived physical fitness. This scale asks participants about general fitness, cardiorespiratory fitness, muscular strength, speed–agility, and flexibility. Response options range from 1 to 5, where 1 is “Very poor” and 5 is “Very good”. The IFIS total score ranges from 5 to 25. This instrument has been validated in women with FM [27].

Fibromyalgia Impact Questionnaire Revised (FIQ-R): This questionnaire assesses the degree of disability or the impact of the disease on daily life in people with FM. It contains 21 items related to symptoms and problems resulting from FM with scores ranging from 0 to 10, where 0 is the absence of problems, and 10 is the existence of problems of the greatest intensity. The possible scores range from 0 to 100, with 100 being the greatest degree of disability due to the disease. However, this tool is composed of various domains: functions (these are the first 9 items and can have a score between 0 and 30), global impact (two items, can take values between 0 and 20), and symptoms (the last 10 items, which can take scores between 0 and 50). The instrument is valid and reliable in women with FM (Cronbach’s alpha = 0.93) [33] and has been validated in its version in Spanish [34].

Risk of falling: This was assessed with the Activities-Specific Balance Confidence Scale (ABC Scale). This scale consists of 16 items in which participants are asked about their confidence in their balance in different situations, with possible responses ranging from 0% to 100% confidence. Thus, the total score ranges from 0% to 100% of total confidence in balance. Percentages below 67% are considered as a high risk of falling [35]. A continuous variable was created with the overall ABC Scale score and another dichotomous variable (Risk of Falling) in which participants were grouped into those who were at risk of falling (Yes: ABC Scale < 67%) and those who were not (No: ABC Scale ≥ 67%). The ABC Scale is validated in its Spanish version [36].

Fear of Falling: This was assessed with the Fall Efficacy Scale-International (FES-I). A questionnaire composed of 16 items in which participants are asked about their level of concern about falls in activities of daily living, with possible responses: 1 (not very concerned) to 4 (very concerned). The FES-I can take values from 16 (not worried at all) to 64 (very worried). Scores equal to or higher than 24 are considered to indicate fear of falling. Two variables were created, a continuous variable with the FES-I score and a dichotomous variable (Fear of Falling) in which participants were grouped into those who were afraid of falling (Yes: FES-I ≥ 24) and those who were not (No: FES-I < 24). This questionnaire is valid and reliable (ICC = 0.96) [37,38,39] and has been validated in its Spanish version [39].

### 2.3. Statistical Analysis

The Kolgomorov–Smirnov test was used to analyze the distribution, followed by the categorical variables of the study. The characterization of the sample was performed through a descriptive analysis by calculating the median and interquartile range (IQR) in the variables: age, BMI, waist:hip ratio, years since diagnosis, years with symptoms, IFIS Score, FIQ-R Score, pain VAS, ABC Scale, FES-I Score, EQ-5D-5L Index, and EQ-5D-5L VAS. In addition, the absolute and relative frequencies presented by the sample in the categorical variables, civil status, employment situation, education level, smoking status, drinking status, fear of falling, and risk of falling, were presented. To test for possible intergroup differences in body composition, physical function, self-perceived physical fitness, quality of life, and the impact of FM (degree of disability due to the disease) as a function of fear of falling and risk of falling, the Mann–Whitney U test was used. Finally, Spearman’s rho was calculated to analyze the correlations between the above-mentioned variables and the total scores on the FES-I and ABC Scale. A *p*-value of *p* < 0.05 with Bonferroni correction was assumed as the significance level. IBM SPSS Statistical v.25 software was used for all analyses.

## 3. Results

Following the results found in the Kolgomorov–Smirnov test, it could not be assumed that the study variables followed a normal distribution.

**Figure 1:**
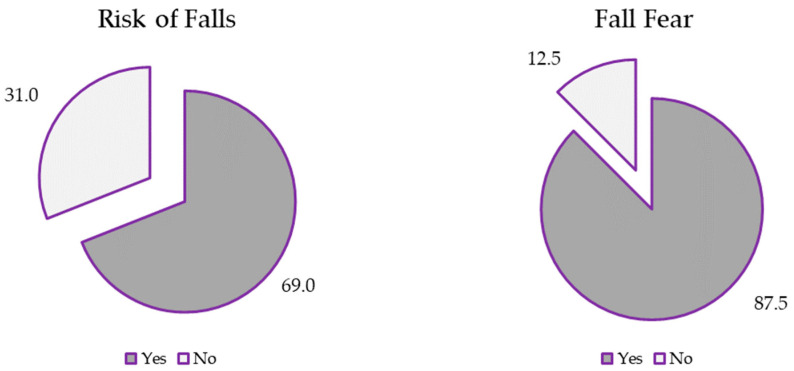
Risk of falls and fall fear proportions in the participants.

The descriptive analysis performed to characterize the sample is shown in Supplementary Table S2. The median age of the participants was 57 years (IQR = 12), with a median of 12 years (IQR = 12) since being diagnosed with FM and 20 years (IQR = 20) since the onset of pathology-related symptoms. The sample had a median number of falls of 1 (IQR = 3) in the previous four months. The impact or degree of disability due to FM, according to the FIQ-R, was 62.1 points (IQR = 28), with a median pain of 6.6 (IQR = 2.0) on the VAS. They had low confidence in their balance, with a median ABC Scale score of 57.7% (IQR = 28.6), almost 10 percentage points below the set point for risk of falling (67%). In addition, they also had elevated fear of falling scores, the median score on the FES-I was 36.5 points (IQR = 15.8), 12.5 points above the cut-off point for fear of falling. Health-related quality of life, according to the EQ-5D-5L Index, was very low, with a median score of 0.170 (IQR = 0.352). Perceived health was also low, with a median score of 50.0 points on the EQ-5D-5L VAS. Finally, 83.3% of the participants had a fear of falling, and 69.0% were at risk of falling (Figure 1).

Although people with a fear of falling (29.3 vs. 25.8, *p* = 0.104. rho: 0.170, *p* = 0.190) and risk of falling (29.3 vs. 28.0, *p* = 0.762. rho: −0.076, *p* = 0.552) showed a higher BMI than people with no fear of falling or risk of falling, no significant differences were found. The same was found for the waist:hip ratio (fall fear: 0.87 vs. 0.86, *p* = 0.326. rho: −0.036, *p* = 0.770; falling risk: 0.87 vs. 0.86, *p* = 0.806. rho: 0.029, *p* = 0.808).

People with a fear of falling had a higher median number of falls in the months prior to testing than people without a fear of falling. However, no significant differences were found (0.5 (2) vs. 1.0 (3.0), *p* = 0.157). A moderate direct correlation was found between the number of falls and the FES-I score (rho: 0.537, *p* < 0.001). Similarly, those at risk of falling had a higher median number of falls than those not at risk of falling (2.0 (4) vs. 1.0 (1.0), *p* = 0.037). An inverse correlation was found between the number of falls and the ABC Scale scores (rho = −0.335, *p* = 0.003).

Table 1 shows the performance of the participants in the physical tests according to whether they had a fear of falling on the FES-I. Better performance in all physical tests was observed in women without fear of falling. However, the differences were not statistically significant. Moderate statistically significant correlations were found between FES-I scores and performance on some physical tests: TUG (rho: 0.406, *p* < 0.001), Dual TUG (rho: 0.419, *p* < 0.001), FSST (rho: 0.363, *p* = 0.001), 30” Sit to Stand test (rho: −0.375, *p* < 0.001) and 5 reps Sit to Stand (rho: 0.356, *p* = 0.001). Individuals with a higher FES-I score had increased times on the TUG, Dual TUG, FSST, and 5 reps Sit to Stand, while they performed fewer repetitions on the 30” Sit to Stand test.

Women without fear of falling presented a higher perception of their physical condition (17 vs. 12, *p* = 0.001), a better health-related quality of life (0.464 vs. 0.159, *p* < 0.001), a better perception of their health (65.0 vs. 49.0, *p* = 0.29) and a lower impact or degree of disability on their life due to FM (36.5 vs. 64.7, *p* < 0.001). Except for the EQ-5D-5L Score (*p* = 0.029), significant differences were found for all other variables. In addition, moderate and statistically significant correlations were found between the FES-I scores and the IFIS, EQ-5D-5L Index, and FIQ-R and all its dimensions (*p* < 0.001) (Table 2).

Although it was found that people with no risk of falling performed better in all physical tests than people with no risk of falling, except for the 30” Biceps Curl test (15 vs. 14, *p* = 0.834), no significant differences were found between groups in most tests, differences were found in the Sit and Reach test, where those with no risk of falling had greater flexibility than those at risk of falling (0.5 vs. −7.5, *p* = 0.002). Although weak to moderate correlations were found between the physical tests and ABC Scale scores, these were not statistically significant (Table 3).

Finally, people who were not at risk of falling showed a higher perception of their physical condition (15 vs. 12, *p* = 0.001), a better health-related quality of life (0.306 vs. 0.134, *p* = 0.004), a better perception of their health (60.0 vs. 45.5, *p* = 0.001) and a lower impact or degree of disability on their life due to FM (36.5 vs. 64.7, *p* = 0.001). In addition, moderate and statistically significant correlations were found between ABC Scale scores and: IFIS, EQ-5D-5L Index, EQ-5D-5L VAS Score, FIQ-R, and all its dimensions (*p* < 0.001) (Table 4).

## 4. Discussion

The current study aimed to explore the association between having high levels of fear of falling and fall risk and variables such as physical function assessed through physical fitness tests, perceived physical function, health-related quality of life, and degree of disability due to FM. The main finding was that women with FM with higher levels of fall risk or fear of falling had significantly lower levels of self-perceived physical function, health-related quality of life, and degree of disability due to FM. Regarding this test, the overall score and all dimensions (function, overall impact, and symptoms) were found to be significant between those FM patients with a fear of falling and/or fall risk and those without them, which confirms hypotheses 1 and 2 of this investigation. Additionally, the physical function tests, fear of falling, and risk of falling were related to the performance in the timed-up and go test, the four step square test, 4 m walking test, 30 s chair to stand test, handgrip strength, and sit and reach test, which confirms hypothesis 3 of this study.

Risk of falling is a crucial variable among women with FM due to the high prevalence of falls [40], risk of falling [20], and high levels of fear of falling [16]. It was suggested that the patterns of falls in people with FM are similar to those observed in middle-aged and older adults, involving factors like dizziness or weakness [40]. Apart from those, physical function issues play a significant role in the fall experiences of people with FM, being especially relevant to those variables related to gait and balance, which are often severely impaired in women with FM [41,42].

The consequences of falling in women with FM are severe. Apart from the evident economic and social burden of falling, experiencing falls often leads to increased fear of falling and the risk of abandoning usual activities that they used to perform before falling. Our results showed that 87% of the women included in the study had higher levels of fear of falling, which was in line with previous research [16,43]. Those with FM who have experienced falls also have a high risk of developing a fear of falling. Thus, they face situations where they desire to maintain their regular activities, but they are aware that doing so may result in falls [40].

The term kinesiophobia emerged to refer to an irrational and debilitating fear of movement due to perceived vulnerability to injury [44]. This fear is often associated with higher pain intensity, disability, and lower quality of life in patients with chronic pain and may mediate the relationship between pain intensity and self-reported disability [45,46]. A previous study explored the associations between fear of falling and kinesiophobia. It was found that there was a strong correlation between the score of the FES-I and the TSK-11, while the association was not observed in healthy women. Furthermore, kinesiophobia was also associated with the degree of disability due to FM, timed-up and go, or the ability to climb stairs [43]. Therefore, assessment of fear of falling in clinical practice is crucial to identify barriers to participation in activities of daily living and physical activity interventions. Furthermore, randomized controlled trials that aim to improve the fear of falling are encouraged. A previous study reduced by ≈20% the fear of falling of women with FM through an 8-week intervention based on exergames [47].

In terms of practical applications, the results of this study are relevant for health care and physical activity professionals to better understand the relationships between fear of falling and risk of falling with actual and perceived limited physical function, poorer quality of life, and a greater degree of disability in women with FM. This will help to ensure that in programs focused on this population, aspects such as reducing the fear of falling and the risk of falls become priority objectives because of their proven relationship with physical function and health-related quality of life. In terms of practical applications in the scientific field, the results of this study provide scientifically supported data for science-based decision-making, helping to understand that limited physical function or poorer perceived physical fitness in women with FM is associated with a greater fear of falling and risk of falling and that these fear and risk is related to lower physical function and perceived physical function.

The current study has several limitations. First, this is a cross-sectional study, so it was not possible to use the results to predict subsequent falls. Second, the sample size was fully comprised of women, so the results cannot be extrapolated to men. Furthermore, the sample was heterogeneous in terms of the degree of disability due to the disease, years since the diagnosis, or physical fitness level. Some variables, such as the age of the participants, could have affected the results. It would be necessary to enlarge the sample in order to implement statistical models to analyze the effects of other variables on the relationships studied in this research. Therefore, further research is needed to explore these associations in more detail and to investigate potential mechanisms underlying these relationships.

## 5. Conclusions

The present research found that women with FM who suffer from fear of falling and are at risk of falling tend to have worse performance in physical function tests. Additionally, women with this pathology who have a fear of falling or fall risk were shown to have worse self-perceived physical fitness, a higher number of falls, poorer quality of life, and a greater degree of disability due to FM.

## Figures and Tables

**Table 1:** Comparisons in physical fitness test performance as a function of fear of falling. Correlations between physical test performance and Fall Efficacy Scale-International.

Variables	Fear of Falling (FES-I)						
	Yes (n = 70)		No (n = 10)				
	Mdn	(IQR)	Mdn	(IQR)	*p*	rho	*p*†
i-Time Up and Go test (s)	5.25	(2.79)	4.69	(2.21)	0.242	0.187	0.0.97
Time Up and Go test (s)	7.70	(3.26)	6.58	(1.21)	0.123	0.406	<0.001 **
Delta Time Up and Go test	9.45	(13.04)	10.24	(7.87)	0.919	0.154	0.172
Dual Time Up and Go test (s)	9.25	(4.77)	7.70	(2.07)	0.078	0.419	<0.001 **
Four Step Square test (s)	7.33	(2.44)	5.48	(2.24)	0.022	0.363	0.001 *
4 m Walking test (s)	2.70	(0.77)	2.26	(0.63)	0.156	0.255	0.024
30 m Walking test (s)	19.15	(7.25)	17.35	(5.31)	0.250	0.170	0.142
6 min Walking test (m)	483	(159)	510	(203)	0.381	−0.168	0.147
30” Sit to Stand test (rep)	10	(5)	12	(2)	0.045	−0.375	0.001 *
Dual 30” Sit to Stand test (rep)	9	(3)	11	(1)	0.035	−0.323	0.003
5 reps Sit to Stand test (s)	13.50	(5.00)	12.38	(2.00)	0.120	0.356	0.001 *
Max Strength Handgrip (kg)	22.5	(6.8)	24.3	(7.0)	0.103	−0.291	0.103
Max Strength/Weight Ratio	0.29	(0.12)	0.39	(0.13)	0.004	−0.345	0.009
30” Biceps Curl test (rep)	14	(6)	17	(6)	0.060	−0.203	0.008
Functional reach test (cm)	34.5	(13.5)	35.3	(9.9)	0.948	0.014	0.902
Sit and Reach (cm)	−2.0	(10.5)	4.0	(16.8)	0.051	−0.320	0.005
Back Scratch (cm)	−4.5	(14)	−0.3	(7.6)	0.087	−0.206	0.066

n (participants); Mdn (median); IQR (interquartile range); *p* (*p*-value from Mann–Whitney U test, Bonferroni correction *p* < 0.007); rho (Spearman rho); *p*† (*p*-value from Spearman rho, Bonferroni correction *p* < 0.007); * (*p* < 0.007); ** (*p* < 0.001); FES-I (Fall Efficacy Scale-International. 16, no concern about falling; 64, severe concern about falling); Yes (FEI-I Score ≥ 24); No (FES-I Score < 24); i (imagined); s (seconds); m (meters); rep (repetitions); Max (maximum); cm (centimeters).

**Table 2:** Comparisons of self-perceived physical fitness, health-related quality of life, and the impact or degree of disability due to fibromyalgia as a function of fear of falling. Correlations between the above variables and Fall Efficacy Scale-International Score.

Variables	Fear of Falling (FES-I)						
	Yes (n = 70)		No (n = 10)				
	Mdn	(IQR)	Mdn	(IQR)	*p*	rho	*p*†
IFIS (Score 5–25)	12	(4)	17	(4)	0.001 *	−0.572	<0.001 **
EQ-5D-5L Index	0.159	(0.291)	0.464	(0.226)	<0.001 **	−0.560	<0.001 **
EQ-5D-5L VAS	49.0	(36.5)	65.0	(20.3)	0.029	−0.295	0.008
FIQ-R Function (Score 0–30)	18.7	(10.3)	8.0	(10.5)	<0.001 **	0.689	<0.001 **
FIQ-R Impact (Score 0–20)	13.0	(6.0)	3.0	(12.0)	0.003 *	0.639	<0.001 **
FIQ-R Symptoms (Score 0–50)	34.5	(10.0)	25.0	(6.3)	0.001 *	0.676	<0.001 **
FIQ-R (Score 0–100)	64.7	(23.3)	36.5	(28.5)	<0.001 **	0.731	<0.001 **

n (participants); Mdn (median); IQR (interquartile range); *p* (*p*-value from Mann–Whitney U test, Bonferroni correction *p* < 0.007); rho (Spearman rho); *p*† (*p*-value from Spearman rho, Bonferroni correction *p* < 0.007); * (*p* < 0.007); ** (*p* < 0.001); FES-I (Fall Efficacy Scale-International. 16, no concern about falling; 64, severe concern about falling); Yes (FEI-I Score ≥ 24); No (FES-I Score < 24). FIQ-R (Fibromyalgia Impact Questionnaire Revised. From 0 to 100 indicating the lowest to highest degree of disability due to the disease); EQ-5D-5L Index (EuroQol. 1: the best state of health. 0: death. Negative values indicate conditions worse than death); EQ-5D-5L VAS (0: the worst health. 100: the best state of health).

**Table 3:** Comparisons in physical test performance as a function of fear of falling. Correlations between physical test performance and Activities-Specific Balance Confidence Scale.

Variables	Risk of Falling (ABC Scale)						
	Yes (n = 58)		No (n = 26)				
	Mdn	(IQR)	Mdn	(IQR)	*p*	rho	*p*†
i-Time Up and Go test (s)	5.27	(2.68)	5.17	(2.94)	0.985	−0.074	0.506
Time Up and Go test (s)	7.93	(3.34)	6.58	(2.06)	0.017	−0.269	0.013
Delta Time Up and Go test	10.31	(13.70)	7.29	(8.60)	0.056	−0.133	0.229
Dual Time Up and Go test (s)	9.90	(4.74)	8.20	(3.08)	0.040	−0.240	0.028
Four Step Square test (s)	7.44	(2.42)	6.52	(2.31)	0.077	−0.256	0.019
4 m Walking test (s)	2.78	(0.91)	2.47	(0.53)	0.025	−0.225	0.042
30 m Walking test (s)	19.59	(6.10)	18.47	(5.16)	0.302	−0.084	0.460
6 min Walking test (m)	467	(143)	505	(201)	0.227	0.136	0.228
30” Sit to Stand test (rep)	10	(4)	11	(4)	0.097	0.257	0.018
Dual 30” Sit to Stand test (rep)	9	(4)	10	(2)	0.444	0.196	0.074
5 reps Sit to Stand test (s)	13.63	(6.00)	12.39	(4.00)	0.026	−0.276	0.012
Max Strength Handgrip (kg)	22.0	(6.5)	23.9	5.1)	0.174	0.218	0.046
Max Strength/Weight Ratio	0.30	(0.13)	0.34	(0.15)	0.336	0.184	0.160
30” Biceps Curl test (rep)	15	(7)	14	(8)	0.834	−0.013	0.904
Functional reach test (cm)	32.8	(13.6)	35.5	(11.5)	0.117	0.083	0.451
Sit and Reach (cm)	−4.0	(12.5)	2.0	(12.3)	0.002 *	0.266	0.016
Back Scratch (cm)	−7.5	(15.0)	0.5	(7.5)	0.003	0.269	0.013

n (participants); Mdn (median); IQR (interquartile range); *p* (*p*-value from Mann–Whitney U test, Bonferroni correction *p* < 0.007); rho (Spearman rho); *p*† (*p*-value from Spearman rho, Bonferroni correction *p* < 0.007); * (*p* < 0.007); ABC (Activities-Specific Balance Confidence Scale. 0: Not confident at all; 100: completely confident); Risk of Falls (Yes: ABC Scale < 67.0; No: ABC Scale ≥ 67.0); i (imagined); s (seconds); m (meters); rep (repetitions); Max (maximum); cm (centimeters).

**Table 4:** Comparisons of self-perceived physical fitness, health-related quality of life, and the impact or degree of disability due to fibromyalgia as a function of risk of falls. Correlations between these variables and ABC Scale.

Variables	Risk of Falling (ABC Scale)						
	Yes (n = 58)		No (n = 26)				
	Mdn	(IQR)	Mdn	(IQR)	*p*	rho	*p*†
IFIS (Score 5–25)	12	(4)	15	(5)	0.001 *	0.446	<0.001 **
EQ-5D-5L Index	0.134	(0.306)	0.306	(0.304)	0.004	0.479	<0.001 **
EQ-5D-5L VAS	45.5	(30.0)	60.0	(25.5)	0.001 *	0.439	<0.001 **
FIQ Function (Score 0–30)	18.7	(8.2)	11.3	(11.6)	0.001 *	−0.578	<0.001 **
FIQ-R Impact (Score 0–20)	13.0	(6.0)	8.5	(12.3)	0.010	−0.412	<0.001 **
FIQ-R Symptoms (Score 0–50)	34.8	(8.4)	26.5	(14.1)	0.002 *	−0.531	<0.001 **
FIQ-R (Score 0–100)	65.5	(22.6)	46.6	(33.7)	0.001 *	−0.580	<0.001 **

n (participants); Mdn (median); IQR (interquartile range); *p* (*p*-value from Mann–Whitney U test, Bonferroni correction *p* < 0.007); rho (Spearman rho); *p*† (*p*-value from Spearman rho, Bonferroni correction *p* < 0.007); * (*p* < 0.007); ** (*p* < 0.001); ABC (Activities-Specific Balance Confidence Scale. 0: Not confident at all; 100: completely confident); Risk of Falls (Yes: ABC Scale < 67.0; No: ABC Scale ≥ 67.0). FIQ-R (Fibromyalgia Impact Questionnaire Revised. From 0 to 100 indicating the lowest to highest impact or degree of disability due to fibromyalgia); EQ-5D-5L Index (EuroQol. 1: the best state of health. 0: death. Negative values indicate conditions worse than death); EQ-5D-5L VAS (0: the worst health. 100: the best state of health).

## Data Availability

The data presented in this study are available on request from the corresponding author. The data are not publicly available due to privacy and ethical restrictions.

## Supplementary Materials

The following supporting information can be downloaded at: https://www.mdpi.com/article/10.3390/jfmk9030174/s1.

## References

[1] Vilarino G.T., Andreato L.V., de Souza L.C., Branco J.H.L., Andrade A. (2021). Effects of Resistance Training on the Mental Health of Patients with Fibromyalgia: A Systematic Review. Clin. Rheumatol..

[2] Sarzi-Puttini P., Giorgi V., Marotto D., Atzeni F. (2020). Fibromyalgia: An Update on Clinical Characteristics, Aetiopathogenesis and Treatment. Nat. Rev. Rheumatol..

[3] Fietta P., Fietta P., Manganelli P. (2007). Fibromyalgia and Psychiatric Disorders. Acta Biomed.-Ateneo Parm..

[4] Işık-Ulusoy S. (2019). Evaluation of Affective Temperament and Anxiety-Depression Levels in Fibromyalgia Patients: A Pilot Study. Braz. J. Psychiatry.

[5] Andrade A., Vilarino G.T., Sieczkowska S.M., Coimbra D.R., Steffens R.d.A.K., Vietta G.G. (2018). Acute Effects of Physical Exercises on the Inflammatory Markers of Patients with Fibromyalgia Syndrome: A Systematic Review. J. Neuroimmunol..

[6] Córdoba-Torrecilla S., Aparicio V., Soriano-Maldonado A., Estévez-López F., Segura-Jiménez V., Álvarez-Gallardo I., Femia P., Delgado-Fernández M. (2016). Physical Fitness Is Associated with Anxiety Levels in Women with Fibromyalgia: The al-Ándalus Project. Qual. Life Res..

[7] Mingorance J.A., Montoya P., Miranda J.G.V., Riquelme I. (2021). An Observational Study Comparing Fibromyalgia and Chronic Low Back Pain in Somatosensory Sensitivity, Motor Function and Balance. Healthcare.

[8] Lee L.K., Ebata N., Hlavacek P., DiBonaventura M., Cappelleri J.C., Sadosky A. (2016). Humanistic and Economic Burden of Fibromyalgia in Japan. J. Pain Res..

[9] Booth J., Moseley G.L., Schiltenwolf M., Cashin A., Davies M., Hübscher M. (2017). Exercise for Chronic Musculoskeletal Pain: A Biopsychosocial Approach. Musculoskelet. Care.

[10] Singh R., Rai N.K., Pathak A., Rai J., Pakhare A., Kashyap P.V., Rozatkar A.R., Mishra S., Mudda S. (2024). Impact of Fibromyalgia Severity on Patients Mood, Sleep Quality, and Quality of Life. J. Neurosci. Rural Pract..

[11] Antunes M., Cruz A.T., Januário P.D.O., Marques A.P. (2022). AB1552-HPR health perception of patients with fibromyalgia in Brazil. Ann. Rheum. Dis..

[12] Jones K.D., Horak F.B., Winters-Stone K., Irvine J.M., Bennett R.M. (2009). Fibromyalgia Is Associated with Impaired Balance and Falls. JCR J. Clin. Rheumatol..

[13] Núñez-Fuentes D., Obrero-Gaitán E., Zagalaz-Anula N., Ibáñez-Vera A.J., Achalandabaso-Ochoa A., López-Ruiz M.D., Rodríguez-Almagro D., Lomas-Vega R. (2021). Alteration of Postural Balance in Patients with Fibromyalgia Syndrome—A Systematic Review and Meta-Analysis. Diagnostics.

[14] Huijnen I.P., Verbunt J.A., Meeus M., Smeets R.J. (2015). Energy Expenditure during Functional Daily Life Performances in Patients with Fibromyalgia. Pain Pract..

[15] Murillo-Garcia A., Villafaina S., Leon-Llamas J.L., Sánchez-Gómez J., Domínguez-Muñoz F.J., Collado-Mateo D., Gusi N. (2021). Mobility Assessment under Dual Task Conditions in Women with Fibromyalgia: A Test-retest Reliability Study. PMR.

[16] Collado-Mateo D., Gallego-Diaz J.M., Adsuar J.C., Domínguez-Muñoz F.J., Olivares P., Gusi N. (2015). Fear of Falling in Women with Fibromyalgia and Its Relation with Number of Falls and Balance Performance. BioMed Res. Int..

[17] Meireles S.A., Antero D.C., Kulczycki M.M., Skare T.L. (2014). Prevalence of Falls in Fibromyalgia Patients. Acta Ortop. Bras..

[18] Russek L., Gardner S., Maguire K., Stevens C., Brown E.Z., Jayawardana V., Mondal S. (2015). A Cross-Sectional Survey Assessing Sources of Movement-Related Fear among People with Fibromyalgia Syndrome. Clin. Rheumatol..

[19] Cigarán-Méndez M., Úbeda-D’Ocasar E., Arias-Buría J.L., Fernández-de-Las-Peñas C., Gallego-Sendarrubias G.M., Valera-Calero J.A. (2022). The Hand Grip Force Test as a Measure of Physical Function in Women with Fibromyalgia. Sci. Rep..

[20] Sarıhan K., Uzkeser H., Erdal A. (2021). Evaluation of Balance, Fall Risk, and Related Factors in Patients with Fibromyalgia Syndrome. Turk. J. Phys. Med. Rehabil..

[21] Vandenbroucke J.P., von Elm E., Altman D.G., Gøtzsche P.C., Mulrow C.D., Pocock S.J., Poole C., Schlesselman J.J., Egger M. (2014). Strengthening the Reporting of Observational Studies in Epidemiology (STROBE): Explanation and Elaboration. Int. J. Surg..

[22] Wolfe F., Rasker J.J., Ten Klooster P., Häuser W., Rasker J.J.J., Klooster P. (2021). Subjective Cognitive Dysfunction in Patients with and without Fibromyalgia: Prevalence, Predictors, Correlates, and Consequences. Cureus.

[23] Rodríguez F.A. (1994). Cuestionario de Aptitud Para La Actividad Física (C-AAF), Versión Catalana/Castellana Del PAR-Q Revisado. Apunt. Med. De L" Esport (Castell.).

[24] Carbonell-Baeza A., Álvarez-Gallardo I.C., Segura-Jiménez V., Castro-Piñero J., Ruiz J., Delgado-Fernández M., Aparicio V.A. (2015). Reliability and Feasibility of Physical Fitness Tests in Female Fibromyalgia Patients. Int. J. Sports Med..

[25] Beauchet O., Fantino B., Allali G., Muir S.W., Montero-Odasso M., Annweiler C. (2011). Timed up and Go Test and Risk of Falls in Older Adults: A Systematic Review. J. Nutr. Health Aging.

[26] Dite W., Temple V.A. (2002). A Clinical Test of Stepping and Change of Direction to Identify Multiple Falling Older Adults. Arch. Phys. Med. Rehabil..

[27] Carlos-Vivas J., Pérez-Gómez J., Delgado-Gil S., Campos-López J.C., Granado-Sánchez M., Rojo-Ramos J., Muñoz-Bermejo L., Barrios-Fernandez S., Mendoza-Muñoz M., Prado-Solano A. (2020). Cost-Effectiveness of “Tele-Square Step Exercise” for Falls Prevention in Fibromyalgia Patients: A Study Protocol. Int. J. Environ. Res. Public Health.

[28] Izquierdo-Alventosa R., Inglés M., Cortés-Amador S., Gimeno-Mallench L., Chirivella-Garrido J., Kropotov J., Serra-Añó P. (2020). Low-Intensity Physical Exercise Improves Pain Catastrophizing and Other Psychological and Physical Aspects in Women with Fibromyalgia: A Randomized Controlled Trial. Int. J. Environ. Res. Public Health.

[29] Andersson M., Moberg L., Svantesson U., Sundbom A., Johansson H., Emtner M. (2011). Measuring Walking Speed in COPD: Test-Retest Reliability of the 30-Metre Walk Test and Comparison with the 6-Minute Walk Test. Prim. Care Respir. J..

[30] Spagnuolo D.L., Jürgensen S.P., Iwama Â.M., Dourado V.Z. (2010). Walking for the Assessment of Balance in Healthy Subjects Older than 40 Years. Gerontology.

[31] Rikli R.E., Jones C.J. (2013). Development and Validation of Criterion-Referenced Clinically Relevant Fitness Standards for Maintaining Physical Independence in Later Years. Gerontol..

[32] Ruiz-Ruiz J., Mesa J.L., Gutiérrez A., Castillo M.J. (2002). Hand Size Influences Optimal Grip Span in Women but Not in Men. J. Hand Surg..

[33] Luciano J.V., Aguado J., Serrano-Blanco A., Calandre E.P., Rodriguez-Lopez C.M. (2013). Dimensionality, Reliability, and Validity of the Revised Fibromyalgia Impact Questionnaire in Two Spanish Samples. Arthritis Care Res..

[34] Salgueiro M., García-Leiva J.M., Ballesteros J., Hidalgo J., Molina R., Calandre E.P. (2013). Validation of a Spanish Version of the Revised Fibromyalgia Impact Questionnaire (FIQR). Health Qual. Life Outcomes.

[35] Ishimoto Y., Wada T., Kasahara Y., Kimura Y., Fukutomi E., Chen W., Hirosaki M., Nakatsuka M., Fujisawa M., Sakamoto R. (2012). Fall Risk Index Predicts Functional Decline Regardless of Fall Experiences among Community-dwelling Elderly. Geriatr. Gerontol. Int..

[36] Montilla-Ibáñez A., Martínez-Amat A., Lomas-Vega R., Cruz-Díaz D., Torre-Cruz M.J.D.l., Casuso-Pérez R., Hita-Contreras F. (2017). The Activities-Specific Balance Confidence Scale: Reliability and Validity in Spanish Patients with Vestibular Disorders. Disabil. Rehabil..

[37] Yardley L., Beyer N., Hauer K., Kempen G., Piot-Ziegler C., Todd C. (2005). Development and Initial Validation of the Falls Efficacy Scale-International (FES-I). Age Ageing.

[38] Kempen G.I.J.M., Todd C.J., Van Haastregt J.C.M., Rixt Zijlstra G.A., Beyer N., Freiberger E., Hauer K.A., Piot-Ziegler C., Yardley L. (2007). Cross-Cultural Validation of the Falls Efficacy Scale International (FES-I) in Older People: Results from Germany, the Netherlands and the UK Were Satisfactory. Disabil. Rehabil..

[39] Lomas-Vega R., Hita-Contreras F., Mendoza N., Martínez-Amat A. (2012). Cross-Cultural Adaptation and Validation of the Falls Efficacy Scale International in Spanish Postmenopausal Women. Menopause.

[40] Rutledge D.N., Martinez A., Traska T.K., Rose D.J. (2013). Fall Experiences of Persons with Fibromyalgia over 6 Months. J. Adv. Nurs..

[41] Peinado-Rubia A., Osuna-Pérez M.C., Rodríguez-Almagro D., Zagalaz-Anula N., López-Ruiz M.C., Lomas-Vega R. (2020). Impaired Balance in Patients with Fibromyalgia Syndrome: Predictors of the Impact of This Disorder and Balance Confidence. Int. J. Environ. Res. Public Health.

[42] Costa I.d.S., Gamundí A., Miranda J.G.V., França L.G.S., De Santana C.N., Montoya P. (2017). Altered Functional Performance in Patients with Fibromyalgia. Front. Hum. Neurosci..

[43] Leon-Llamas J.L., Murillo-Garcia A., Villafaina S., Domínguez-Muñoz F.J., Morenas J., Gusi N. (2022). Relationship between Kinesiophobia and Mobility, Impact of the Disease, and Fear of Falling in Women with and without Fibromyalgia: A Cross-Sectional Study. Int. J. Environ. Res. Public Health.

[44] SH K. (1990). Kinesiophobia: A New View of Chronic Pain Behavior. Pain Manag..

[45] Luque-Suarez A., Martinez-Calderon J., Falla D. (2019). Role of Kinesiophobia on Pain, Disability and Quality of Life in People Suffering from Chronic Musculoskeletal Pain: A Systematic Review. Br. J. Sports Med..

[46] Varallo G., Scarpina F., Giusti E.M., Cattivelli R., Guerrini Usubini A., Capodaglio P., Castelnuovo G. (2021). Does Kinesiophobia Mediate the Relationship between Pain Intensity and Disability in Individuals with Chronic Low-Back Pain and Obesity?. Brain Sci..

[47] Collado-Mateo D., Dominguez-Muñoz F.J., Adsuar J.C., Merellano-Navarro E., Gusi N. (2017). Exergames for Women with Fibromyalgia: A Randomised Controlled Trial to Evaluate the Effects on Mobility Skills, Balance and Fear of Falling. PeerJ.

