# Supplementary material for: Physical Function, Self-Perceived Physical Fitness, Falls, Quality of Life and Degree of Disability According to Fear and Risk of Falling in Women with Fibromyalgia
Source: J Funct Morphol Kinesiol. 2024 Sep 23;9(3):174. doi: 10.3390/jfmk9030174 (PMC11432931; doi:10.3390/jfmk9030174)
Supplement: Supplementary file 1 [file jfmk-09-00174-s001.zip › Table S2. Descriptive Analysis Falls.pdf]

Table S2. Characterisation of the sample.

Variables	Total (n=84)	
	Median	(IQR)
Age (Years)	57.0	(12.0)
BMI (kg/m2)	28.6	(10.3)
Waist:Hip ratio	0.86	(0.11)
Years since diagnosis	12.0	(14.0)
Years with symptons	20.0	(20.0)
IFIS (Score 0-25)	13.0	(4.0)
Falls (number)	1.0	(3.0)
FIQ-R (Score 0-100)	62.1	(28.0)
VAS Pain (Score 0-10)	6.6	(2.0)
ABC Scale (Score 0-100)	54.7	(28.6)
FES-I (Score 16-64)	36.5	(15.8)
EQ-5D-5L Index	0.170	(0.352)
EQ-5D-5L VAS (Score 0-100)	50.0	(31.0)
Civil Status	n	%
Single	21	25.0
Married	53	63.1
Separated	6	7.1
Widower	3	3.6
Employment situation		
Active	36	42.9
Retired	26	31.0
Unemployed	20	23.8
Househousband/wife	2	2.4
Education level		
Primary Education	32	38.1
Vocacional Training	13	15.5
Secondary Education or Baccalaureate	22	26.2
University	17	20.2
Smoking Status		
Smoker	9	10.7
No Smoker	75	89.3
Drinking Status		
Frequently or Very Frequently	12	14.3
Occasionally	15	17.9
Never o rarely	57	67.9
Fall Fear (FES-I)		
Yes	70	87.5
No	10	12.5
Risk of Falls (ABC Scale)		
Yes	58	69.0
No	26	31.0

n (Participants); Mdn (Median); IQR (Interquartilic Range); BMI (Body Mass Index); IFIS (International Fitness Scale. 5: the worst self-reported fitness; 25: the best self-reported fitness); Falls (Number of falls in the last 4 months); FIQ-R (Fibromyalgia Impact Questionnaire Revised. From 0 to 100 indicating the lowest to highest impact); VAS (Visual Analogic Scale); ABC (Activities-Specific Balance Confidence Scale. 0: Not confident at all; 100: completely confident); FES-I (Fall Efficacy Scale-International. 16, no concern about falling; 64, severe concern about falling); EQ-5D-5L Index (EuroQol. 1: the best state of health. 0: death); EQ-5D-VAS (0: the worst health. 100: the best state of health); Fall Fear (Yes: FES-I ≥24; No: FES-I <24); Risk of Falls (Yes: ABC Scale <67.0; No: ABC Scale ≥ 67.0).
